# A Gamified Real-time Video Observed Therapies (GRVOTS) Mobile App via the Modified Nominal Group Technique: Development and Validation Study

**DOI:** 10.2196/43047

**Published:** 2023-03-07

**Authors:** Siti Aishah Abas, Nurhuda Ismail, Yuslina Zakaria, Ismassabah Ismail, Nurul Hidayah Mat Zain, Siti Munira Yasin, Khalid Ibrahim, Asmah Razali, Mas Ahmad Sherzkawee Mohd Yusoff, Norliza Ahmad, Thilaka Chinnayah

**Affiliations:** 1 Department of Public Health Medicine Faculty of Medicine Universiti Teknologi MARA Sungai Buloh Campus Sungai Buloh Malaysia; 2 Department of Pharmacology Faculty of Pharmacy Universiti Teknologi MARA Puncak Alam Campus Puncak Alam Malaysia; 3 Centre of Foundation Studies Universiti Teknologi MARA Cawangan Selangor, Kampus Dengkil Dengkil Malaysia; 4 Faculty of Computer and Mathematical Science Universiti Teknologi MARA Cawangan Melaka, Kampus Jasin Jasin Malaysia; 5 Tuberculosis and Leprosy Control Sector Disease Control Division Ministry of Health Putrajaya Malaysia; 6 TB/Leprosy Disease Unit Selangor State Health Department Shah Alam Malaysia; 7 TB/Leprosy Disease Unit Negeri Sembilan State Health Department Seremban Malaysia

**Keywords:** video directly observed therapy, VDOT, mobile health, mHealth, tuberculosis, medication adherence, directly observed therapy, video-observed therapy, mobile app, mobile health app, gamification

## Abstract

**Background:**

The success rate of tuberculosis (TB) treatment in Malaysia remains below the recommended World Health Organization target of 90% despite the implementation of directly observed therapy, short-course, a physical drug monitoring system, since 1994. With increasing numbers of patients with TB in Malaysia defaulting on treatment, exploring another method to improve TB treatment adherence is vital. The use of gamification and real-time elements via video-observed therapies in mobile apps is one such method expected to induce motivation toward TB treatment adherence.

**Objective:**

This study aimed to document the process of designing, developing, and validating the gamification, motivation, and real-time elements in the Gamified Real-time Video Observed Therapies (GRVOTS) mobile app.

**Methods:**

The modified nominal group technique via a panel of 11 experts was used to validate the presence of the gamification and motivation elements inside the app, which were assessed based on the percentage of agreement among the experts.

**Results:**

The GRVOTS mobile app, which can be used by patients, supervisors, and administrators, was successfully developed. For validation purposes, the gamification and motivation features of the app were validated as they achieved a total mean percentage of agreement of 97.95% (SD 2.51%), which was significantly higher than the minimum agreement score of 70% (*P*<.001). Further, each component of gamification, motivation, and technology was also rated at 70% or more. Among the gamification elements, fun received the lowest scores, possibly because the nature of serious games does not prioritize the fun element and because the perception of fun varies by personality. The least popular element in motivation was relatedness, as stigma and discrimination hinder interaction features, such as leaderboards and chats, in the mobile app.

**Conclusions:**

It has been validated that the GRVOTS mobile app contains gamification and motivation elements, which are intended to encourage medication adherence to TB treatment.

## Introduction

More than 10 million cases of tuberculosis (TB) are reported each year globally [1]. In Malaysia, from 2010 to 2015, TB cases increased from 68.4 to 79.6 cases per 100,000 people [2]. In addition, according to a model projection, the observed and projected TB incidence in Malaysia will reach 300,000 cases in 2030 [3].

In Malaysia, directly observed therapy, short-course (DOTS) is a method to ensure medication compliance by having a trained health care worker or other designated individual provide the prescribed TB drugs and watch the patient take every dose. DOTS, which was implemented in 1994, has resulted in a treatment success rate from 76% in 2013 to 81% in 2017 [2]. However, this rate has remained below the recommended World Health Organization target of 90%. In addition, there is an increase in the prevalence of TB treatment default in Malaysia, which has ranged from 4% in 2010 to 4.8% in 2015 and to 5.6% in the latest study [4]. The increasing number of TB cases indicates that there are still issues and challenges that need to be addressed at all levels.

Specifically, the problems currently facing DOTS can be categorized according to the 3 main stakeholders. For patients with TB, the compulsory need for daily DOTS monitoring results in stigma by the public and absence from home or work responsibilities [5-7]. Health workers and policy makers are hesitant to fully implement DOTS, as there are inadequate human resources, increased TB management costs, less participation from lower management, and a lack of public awareness [8,9].

To address these challenges, the World Health Organization recommends the use of digital technologies to promote TB medication adherence [10]. Video directly observed therapy (VDOT) was introduced to replace physical DOTS and has proven to significantly reduce the cost of managing TB, improve patients’ access to doctors, and be less disruptive to patients’ work and family life [8,11-13]. According to many studies, VDOT is a more cost-effective method that significantly increases patient treatment adherence compared to conventional DOTS [14-18].

Although data on VDOT are becoming increasingly robust, the system has yet to be rigorously evaluated within low- and middle-income countries, especially regarding its feasibility [19]. This is quite worrying, as the implementation of VDOT requires complete access to hardware and internet connectivity, which some countries cannot afford [10]. However, in Malaysia, the number of smartphone users is growing and expected to reach over 33 million by 2024, with an 87.36% smartphone penetration, which suggests that VDOT can be implemented in Malaysia [20].

Making VDOT available via mobile apps could make drug monitoring more convenient and effective [21]. However, with the large numbers of mobile health apps in existence, the problem is often about the sustainability of their use [22]. As a solution, integrating gamification elements inside mobile apps can positively impact health and well-being, improve health behaviors and patient engagement, decrease health care use, and empower patients to self-manage their disease [23,24]. In addition, the use of real-time elements such as virtual reality and augmented reality has proven to increase learning effectiveness and behavior modification, correct medication identification, correct self-administration of medication, and support patient counseling practices [25-27].

Thus, in our Gamified Real-time Video Observed Therapy (GRVOTS) mobile app, the integration of gamification and real-time elements is expected to increase patient motivation. The purpose of this study was to validate the gamification, motivation, and real-time element in GRVOTS, a mobile app for VDOT, from the perspective of the service provider (expert review).

## Methods

The mobile app prototype was developed from February 2021 to May 2022. The developmental process as a whole, including content and prototype development as well as content validation (nominal group technique [NGT]), was performed using the design science research process model [28].

### Development

Content development of the prototype involved a few stages of literature review, mapping, and justification of the new framework. From the literature review, we identified 3 frameworks that could be used as features of app: gamification framework, video reality and motivation framework, and technology feature framework. The gamification component, as the foundation of the proposed gamified mobile app GRVOTS, will be based on the validated framework for the gamification of diabetes self-management called The Wheel of Sukr [29]. The framework consists of 8 components: fun, esteem, growth, motivation, sustainability, socializing, self-representation, and self-management. For the video reality and motivation framework, we used the attention, relevance, confidence, and satisfaction (ARCS) model of motivational design [30]. In terms of the mapping procedure, the ARCS model of motivational design can be combined with the gamification elements to foster motivation [31]. The dynamic nature of gamification, such as self-management, self-representation, and fun, can be equal to satisfaction in the ARCS model. Further, elements of gamification, such as esteem, reward, growth, and socializing, can be equal to the components of confidence in the same model. Subsequently, these 2 frameworks can be integrated where gamification elements are added to the categories of confidence and satisfaction that are based on the ARCS model. Table 1 shows the categories and subcategories of the proposed model in matrix form.

**Figure 1:**
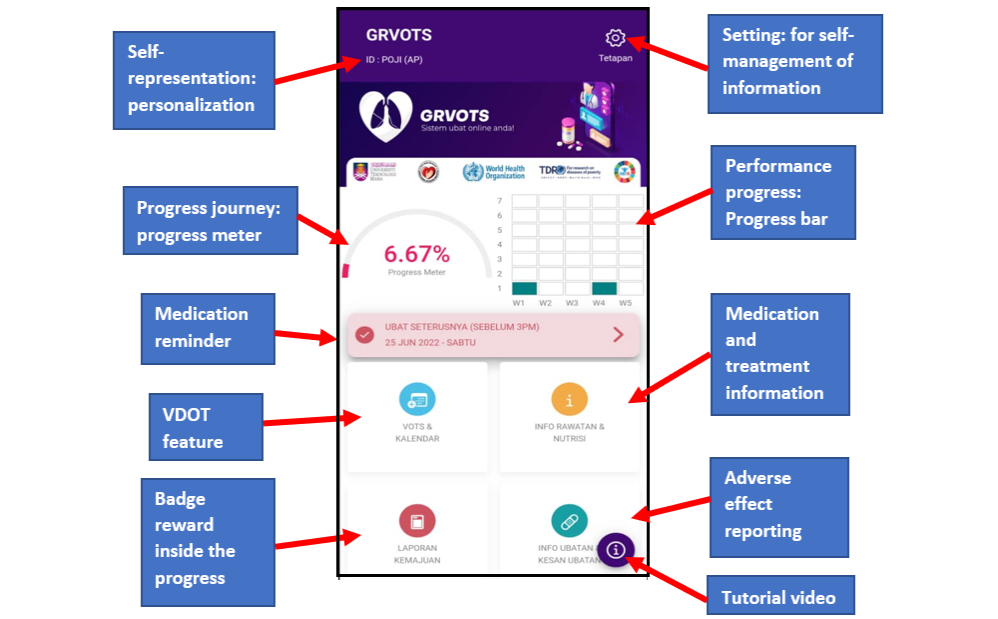
The screenshot from the apps and justification of the related element. GRVOTS: Gamified Real-time Video Observed Therapies; VDOT: video directly observed therapy.

Game dynamics can improve user desire and motivation by establishing rules that encourage users to explore and learn about the apps [32]. Figure 1 shows a screenshot of the main function of the GRVOTS mobile app and its relation to our intended gamification, motivation, and real-time elements from the framework.

**Figure 2:**
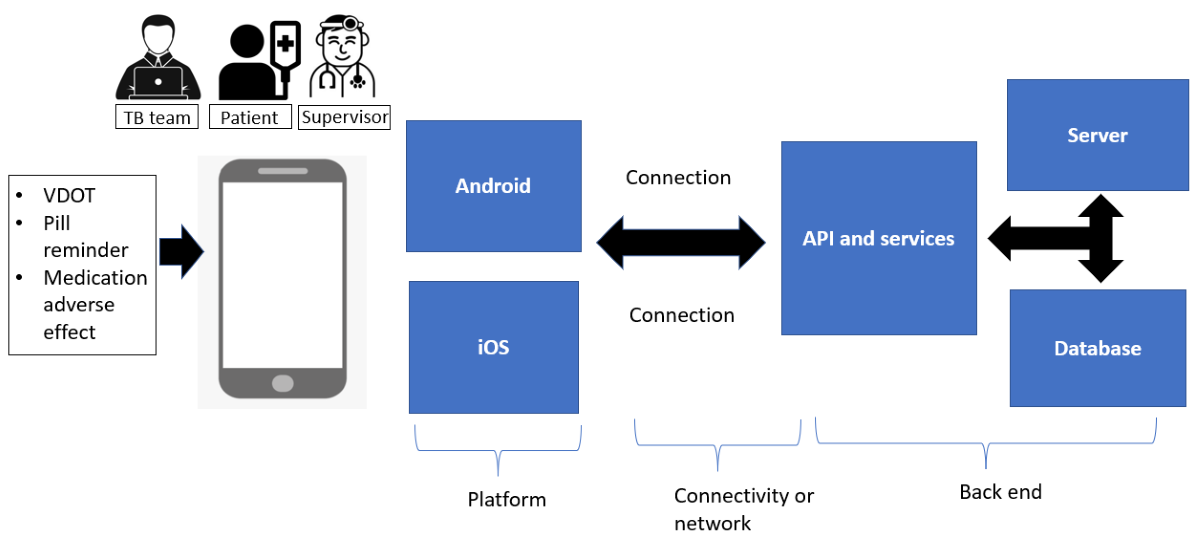
Systematic architecture of GRVOTS. Data from patients were collected through the apps and process with predesigned rules. API: application programming interface; GRVOTS: Gamified Real-time Video Observed Therapies; iOS: iPhone Operating System; TB: tuberculosis; VDOT: video directly observed therapy.

The GRVOTS mobile app is designed for 3 users—patients, supervisors, and administrators—where they interact with one another via the internet. All data inputted by the patients will be automatically collected by the server and viewed by the specific supervisor (health care worker) and TB management team to help them with clinical interventions. The proposed model presented in Figure 2 is based on the development of mobile apps for smartphones only.

**Table 1:** Matrix of the ARCS^a^ model of motivational design and The Wheel of Sukr gamification model.

Model technique	Category			
	Attention	Relevance	Confidence	Satisfaction
ARCS (motivation)	Active participation	Link previous experience (motive matching) Perceive with future usefulness (motive matching)	Self-growth Learning requirement Success Opportunities Personal responsibilities	Reward Immediate application
The Wheel of Sukr (gamification)	—^b^	—	Esteem Reward Growth Socializing Sustainability	Self-management Self-representative Fun

^a^ARCS: attention, relevance, confidence, and satisfaction.

^b^Not applicable.

### Content Validation via the NGT (Expert Review)

The NGT is a structured variation of a small-group discussion to reach consensus. Through the agreement of the description of the elements, the NGT was used in this research as a validation tool to evaluate the presence of the gamification and motivation components intended to be used in the app.

### Sample Size

The NGT is a small-group technique suited to panel sizes of more than 10 people [33,34]. Therefore, there were 11 experts involved in this NGT session. A panel of experts was involved to validate the gamification elements in the GRVOTS app using physical meetings in 3 different settings.

### Study Population Flow

**Figure 3:**
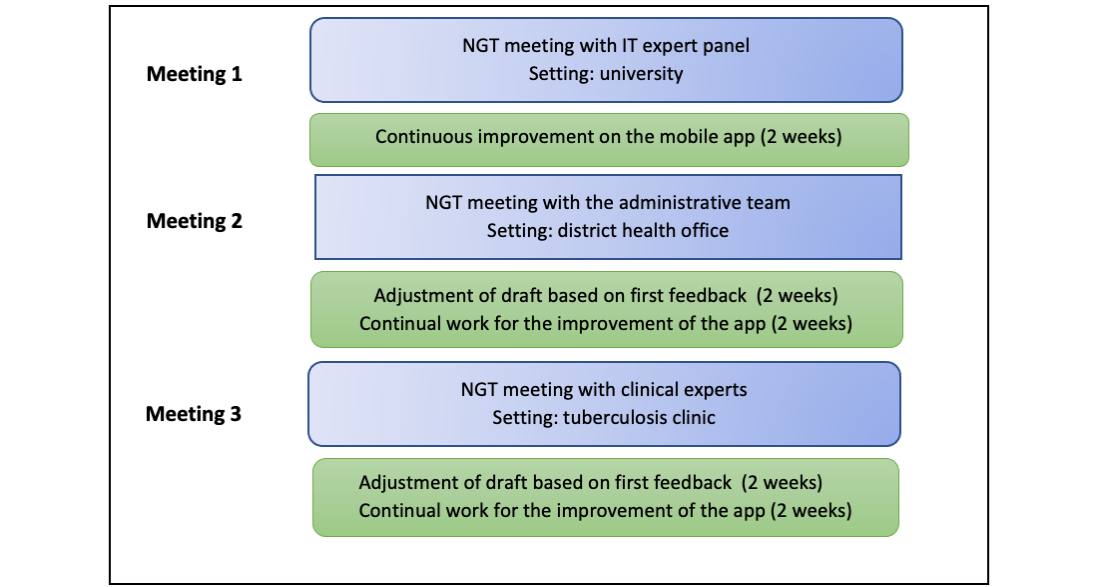
Flow of the modified nominal group technique (NGT) meetings.

This study was conducted iteratively in 3 meetings for a more comprehensive evaluation, as illustrated in Figure 3.

The criteria of the experts involved in the group were different according to each group. For the first group, IT experts were experienced and involved in mobile app development for at least 2 years and well versed in the gamification features of mobile apps. The second and third groups were composed of administrative and health care workers who were directly involved in managing patients with TB in the outpatient environment, respectively.

### Study Settings

The 3 NGT meetings were performed in 3 settings: at the university for the meeting with the IT expert panel, at the district health office for the meeting session with the administrative team, and at the TB clinic for the meeting with clinical experts.

### Instrument for NGT

The instrument used in the NGT was a questionnaire, and the items were generated from a literature review based on 3 existing models as per the previous mapping. These models were developed into a panel expert checklist, and the questionnaire had 2 parts: Part A asked about the gamification-motivation–rea-time theory, The Wheel of Sukr [35], and the ARCS model, whereas Part B concerned the technology features by Anderson et al [36].

### Implementation of NGT

The implementation of NGT involved experts who were selected according to the scope of the study. The workshop was conducted in a face-to-face meeting by a moderator [34]. The NGT workshop lasted approximately 2 hours. Before the workshop, the experts were given a week to use the app. Some of them experienced the task as a supervisor and some as a patient. They were required to send 3-4 VDOT videos. Table 2 shows the basic steps to carry out the NGT process, and Table 3 shows the 5 steps of data analysis for the NGT.

The data analysis process for the NGT was based on the percentage of agreement where an element is accepted when the percentage of agreement is 70% or more [37]. The 1-sample, 2-tailed *t* test was used to determine whether the mean (SD) percentage of agreement result on the gamification and motivation elements in this app was significantly higher than the percentage of agreement of 70%, with a level of significance (α error) less than .05. The software used at this stage was SPSS (version 28.0; IBM Corp) and Microsoft Excel.

**Table 2:** Steps of the specific guide to implement the nominal group technique session.

Step	Details
1. Introduction to problem statement and explanation	Moderator will brief the participants regarding the flow of the session.
2. Silent generation of ideas in writing	According to the checklist and GRVOTS^a^ mobile app, participants were asked to answer the questionnaire using a Likert scale.
3. Round robin phase: sharing ideas	Participants were invited to share their answers in the round robin manner.
4. Discussion of ideas	Participants were asked to justify the need for the least prominent gamification and virtual reality elements in the GRVOTS mobile app prototype.
5. Voting and ranking	The voting was done by marking responses on a Likert scale from 1 (totally disagree) to 5 (totally agree). The calculation was done, and the elements were ranked accordingly.

^a^GRVOTS: Gamified Real-time Video Observed Therapies.

**Table 3:** The steps of data analysis for the nominal group technique (NGT) steps.

Step	Activity
1	Ensuring the number of participants (experts) involved in the study
2	The formation and calculation of score value is based on the template data analysis of the NGT
3	Convert score values into percentage form to obtain the percentage of agreement: 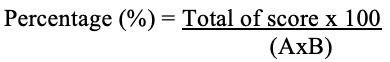 where A=the total number of experts and B=Likert scale used, ie, 5 points
4	Determine the acceptance of components and elements based on the percentage of agreement
5	Determine the positions of the elements according to the percentage of agreement

### Ethics Approval

Ethical approval was obtained from the Universiti Teknologi MARA Ethical Board and Medical Research and Ethics Committee, Malaysia: NMRR-21-1016-58994 (IIR).

No informed consent was taken in this study as the data were only retrieved retrospectively from the database and no identifiers were collected for this study.

## Results

Mobile app development with the integration of gamification and real-time elements was performed via a literature review, and the content validation of the component was performed via the modified NGT.

### Prototype

The final beta version of the mobile app used Android as the platform. The prototype was designed to be used by 3 users—patients, supervisors, and TB managers—and each user had a different role in the program. This mobile app’s main function is to provide patients with a way to record daily DOTS intake via video (VDOT) as well as medication side effect reporting. After patients log in, they are directed to set up their personal profiles and learn how to use the VDOT medication reminder, which is the main activity. After each VDOT session is uploaded, it is followed by pop-up motivational quotes as well as the movement of the progress meter indicator.

The accumulated points collected from the progress meter will be translated into badges in the progress report theme. Subsequently, a daily pop-up message will also be the main reminder for the next medication, and patient will be asked if they noticed any adverse reactions to previous medication, with a selection of options concerning their symptoms. The ability to report adverse effects gives patients access to their own medication diaries, which can be reviewed during medical visits. Throughout the TB treatment journey, the progress report theme will help patients track their journey and redeem the internal and external rewards offered.

Every VDOT report and any side effects noted by patients will be verified by the supervisor as a feedback interaction. This feature enables supervisors to regularly check and understand the patients’ progress as well. In addition, patients can always go to other main theme of “knowledge” to continuously learn more about TB treatment and nutrition.

**Figure 4:**
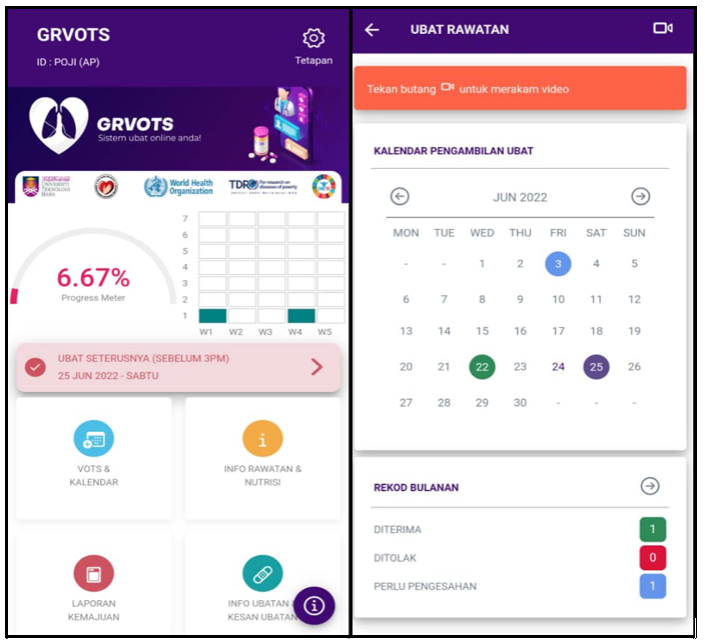
Screenshots of the GRVOTS app for patients. GRVOTS: Gamified Real-time Video-Observed Therapies.

**Figure 5:**
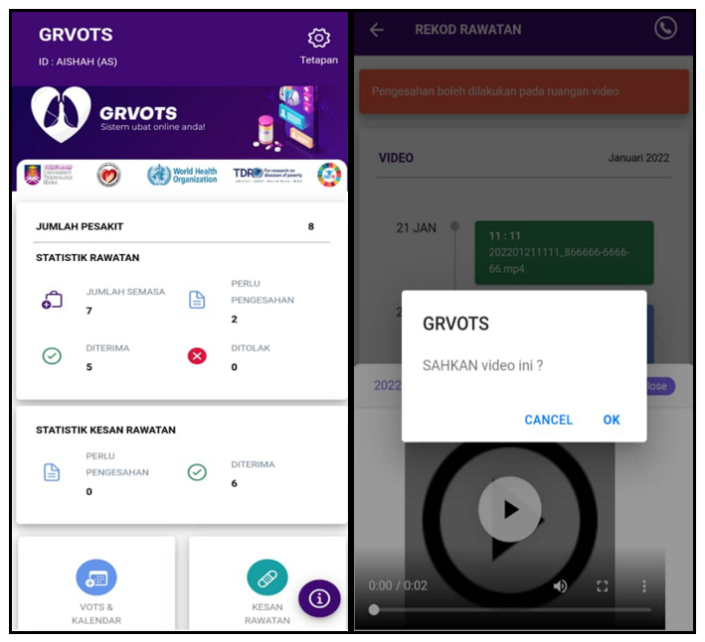
Screenshots of the GRVOTS app for supervisors to monitor patients. GRVOTS: Gamified Real-time Video-Observed Therapies.

The app provides different users with access to different functions and main menus. In the patients’ main menu, there are 4 main themes for VDOT. Information regarding the app, progress report, and side effect reporting is shown in Figure 4. For the supervisors’ main menu, there are 2 menus, one to validate the VDOT report and another to validate the side effects, as shown in Figure 5.

### NGT Output for Content Validation

A panel of 11 experts were involved in the modified NGT, including 2 gamification experts, 2 public health experts, 1 chest physician, 2 medical officers, 3 medical assistants, and 1 staff nurse. They were asked to rank the app’s elements based on the expert panel checklist. Tables 4-6 show the results and rankings of gamification, motivation, and technology feature elements via the NGT.

The total percentage of agreement among experts was 97.95% (SD 2.51%), which was significantly higher than the minimum of 70%, with a difference of 27.947% (95% CI 26.74%-29.15%; *P*<.001). The *t* test confirmed the validity of all gamification and motivation components in the app.

**Table 4:** Results of the nominal group technique for gamification elements.

No	Main element	Explanation element	Percentage of agreement, mean (SD)	Acceptance result	Ranking
1	Self-management	This app has a dashboard with a progress meter, medication reminders, and a VDOT^a^ feature to help patients self-manage their medication. This app has adverse effect reporting to help patients self-manage their reactions to medications.	98 (2.22)	Accepted	4
2	Self-representative (avatar)	This app has a personalized name on the dashboard along with the roles of the user.	97 (0.00)	Accepted	6
3	Fun	This app has a progress meter, a progress bar, motivational quotes, and badge rewards that add a fun element for the patient.	96 (0.8)	Accepted	8
4	Esteem	This app has a progress meter, a progress bar, motivational quotes, and a feedback mechanism aimed at boosting patients’ self-esteem.	99 (1.386)	Accepted	3
5	Growth	This app has a feedback mechanism that helps patients with self-growth. This app empowers patients’ self-growth with medication information.	100 (0.00)	Accepted	1
6	Sustainability (trigger)	This app has medication reminders aimed at the sustainability of its use.	97 (0.00)	Accepted	7
7	Motivation	This app has a progress meter, a progress bar, and badge rewards that boosts patient motivation.	98 (2.358)	Accepted	5
8	Socializing	This app has a feedback mechanism that enables patient-supervisor communication.	100 (0.00)	Accepted	2

^a^VDOT: video directly observed therapy.

**Table 5:** Results of the nominal group technique for motivation elements.

No	Main element	Explanation element	Percentage of agreement, mean (SD)	Acceptance result	Ranking
1	Attention	This app has medication reminders, a VDOT^a^ feature, and adverse effect reporting to hold patients’ attention and achieve active participation. This app has a progress meter that alerts patients regarding the status of their performance. This app has a performance indicator (progress bar) that alerts patients about medication compliance over time.	99 (1.869)	Accepted	3
2	Relevance	This app has a VDOT feature that links patients’ current remote and previous in-person experience of taking medication.	98 (0.00)	Accepted	4
3	Confidence	This app has motivational quotes to boost patient confidence.	100 (0.00)	Accepted	1
4	Satisfaction (achievement)	This app has a VDOT feature, adverse effect reporting, a progress meter, a progress bar, motivational quotes, and badge rewards that help patients feel satisfied with their daily medication compliance.	99 (1.361)	Accepted	2

^a^VDOT: video directly observed therapy.

**Table 6:** Results of the nominal group technique for technology feature elements.

No	Main elements	Explanation element	Percentage of agreement, mean (SD)	Acceptance result	Ranking
1	Automation	This app has automated medication reminders, pop-up notifications, and reminders to both the patient and supervisor that made it easy to use.	97 (0.635)	Accepted	5
2	Ease of use (automation)	This app has automated pop-up notifications to remind the supervisor to approve the adverse effect reports.	100 (0.00)	Accepted	1
3	Real-time feature	This app has a real-time stamped VDOT^a^ feature.	89 (0.00)	Accepted	7
4	Self-guided video	This app has a self-guided VDOT tutorial video that can benefit the patient. This app has self-guided adverse effect symptom choices that can help patients identify symptoms. This app has a self-guided feature to provide the patient with knowledge regarding nutrition and medication. This app has a good tutorial on how to use the app.	99 (1.361)	Accepted	3
5	Aesthetic	This app has good aesthetic features.	97 (0.00)	Accepted	6
6	Navigation	This app has a good flow of navigation from one page to another.	100 (0.00)	Accepted	2
7	Tactile feedback	This app has good tactile or haptic feedback.	98 (0.00)	Accepted	4

^a^VDOT: video directly observed therapy.

## Discussion

This comprehensive GRVOTS mobile app was developed based on the integration of gamification and real-time motivational elements of autonomy, competence, relatedness, attention, relevance, self-control, confidence, and satisfaction. These components were successfully validated and significant.

### Mobile App Developed

Our GRVOTS mobile app was able to motivate patients toward medication adherence using the ARCS model of motivation, which was translated via its gamification and motivation features. First, the interactive VDOT feature can stimulate the patient’s feelings of attention and arousal. Second, by linking the previous experience of medication intake physically with the experience of using GRVOTS, the element of “relevance” can also be instilled in the patient. When patients perceive a high sense of relatedness, they are more likely to exhibit higher engagement with a program [38]. By providing goals along the journey toward medication adherence, the app can instill confidence and self-belief, especially when the patients can digitally see their previous VDOT report and a progress meter of treatment success. As they use GRVOTS to monitor their medication intake, patients experience more freedom of choice and self-control, and the results of treatment adherence will subsequently reinforce the app’s value. The last element of “satisfaction” can also be realized when patients comply with the use of GRVOTS and receive rewards in the form of badges and progress meters.

### Content Validation via the Modified NGT

The results of the modified NGT showed that all the components obtained from the literature and related model were validated by the experts during the NGT session, as shown in Tables 4-6. Based on the results, the elements were prioritized based on the percentage of acceptance. Considering these rankings, some of the GRVOTS functions were improved to provide a better mobile app for the next pilot study.

According to the results for the gamification elements, the growth element received the highest scores, whereas fun received the lowest scores. By definition, “fun” aspects within the gamification elements in this GRVOTS app are exemplified by a progress bar, a progress meter, inspiration quotes, and badge rewards. The fun element was voted as a less visible element in this app, perhaps due to many factors, such as the perception of fun, which varies for different people, as well as the nature of serious games, which does not prioritize the fun element. A number of studies have described that the perception of gamification features differs according to the gamer type, gender, and personality of the player. For instance, extroverts like rewards and leaderboards, which appear to be more entertaining, but introverts prefer badges and feedback [39-41].

Health apps are also considered serious games that are played for purposes other than pure entertainment [42]. In this case, having a fun component is not a priority. According to a study regarding serious games, other elements, such as explicit learning tasks, instruction, and support built into the game or added by teachers, may be more important than having fun while playing [43].

For the motivation elements of the ARCS model, the highest ranked element was confidence and the lowest was relevance. It is suggested that when patients perceive a high sense of relatedness, they are more likely to exhibit higher engagement with the program [38]. In mobile apps, relatedness is seen as a feature of leaderboards or chats that encourages engagement and collaboration to achieve a particular objective [41]. However, in this GRVOTS mobile app, the relevance element is evidenced by allowing daily communication between patients and supervisors via the VDOT and adverse event reporting components without the leaderboard or chat features. This is because most of our patients with TB refused to disclose their condition and communicate with other patients due to the stigma associated with the disease.

For the technology feature elements, the highest ranked was ease of use and the lowest was real-time features. There are 2 type of VDOT: live VDOT, also known as synchronous VDOT, in which patients and providers interact in real time [12,44]; as well as asynchronous technologies that record, upload, and digitally store videos for future review [11,19,45]. Synchronous VDOT has the advantage of human interaction but is not a feasible option, as patients and workers need to find time to meet. Asynchronous VDOT is more flexible but can be manipulated easily by sending the same recorded video. Thus, apps with real-time features ensure the originality of the video and simultaneously generate a greater degree of user engagement [25-27]. Although the real-time feature in our app provides details concerning the time and date of the video, feedback from the experts indicated that we should time stamp the videos so that they can be identified more easily later. Since users found it challenging to determine if their VDOT session was successfully uploaded or not, the addition of an upload bar was also recommended.

### Strengths and Limitations

The GRVOTS mobile app can benefit users in many ways. For patients with TB, the app can help patients gain self-control, boost their self-esteem, and motivate them to take their medications. For health care workers, this app made it easier for TB system management to detect DOTS defaulters and manage them accordingly. As a portable device, mobile apps enable monitoring that can be done anywhere, saving money and time and boosting patient engagement with the DOTS program. In summary, this app can also initiate a patient self-care system and reduce dependency on health care providers such as doctors and nurses. The limitation of this study is that the mobile app prototype is only being developed for the Android platform because of time and logistics. The app does not have a virtual reality feature, and improvements are needed in the future. This study conducted the validation of the gamification and motivation elements only from the perspective of the experts and not from the perspective of patients, which may limit the review’s validity; an analysis regarding its usability among patients will be conducted in the future.

### Recommendation

In the future, GRVOTS should also be available on other platforms, especially the iPhone Operating System. The language options should also include English, Chinese, and Tamil, as these languages are frequently used in multiethnic communities in Malaysia. The use of various languages will expand the benefits to more users. This will enhance knowledge transfer and improve users’ understanding. Next, a usability study to access the user experience will be conducted, followed by an effectiveness study via a single-arm intervention study, in which patients will use the app for DOTS during intensive phase up to 2 months monitoring, followed by an assessment at 3 time intervals to evaluate their medication adherence, motivation, and the usability of the app.

### Conclusion

More comprehensive and efficient TB system management via VDOT mobile app monitoring is a way to improve patient treatment adherence. According to the literature review, gamification elements can motivate patients; thus, by integrating the uniqueness of gamification and motivation elements in an app, gamification will increase patient motivation, ensure the sustainability of use, and ultimately increase patient adherence. In addition, our GRVOTS mobile app connects up to 3 users (eg, patients, supervisors, and administrators) remotely and enable DOTS monitoring to be performed from anywhere. Based on the study findings, the GRVOTS mobile app has been validated by the expert panel as having the intended elements of gamification, real time, and motivation. Next, a usability study of the GRVOTS mobile app will be conducted to measure the user experience among patients, followed by a single-arm intervention study to assess the app’s effectiveness in increasing patient motivation and medication adherence in TB treatment.

## Abbreviations

ARCS: attention, relevance, confidence, and satisfaction
DOTS: directly observed therapy, short-course
GRVOTS: Gamified Real-time Video Observed Therapies
NGT: nominal group technique
TB: tuberculosis
VDOT: video directly observed therapy

## Data Availability

All data supporting the study findings are within the manuscript. Additional details information and raw data are available from the corresponding author on reasonable request.
